# Hypoxic pre-conditioning increases the infiltration of endothelial cells into scaffolds for dermal regeneration pre-seeded with mesenchymal stem cells

**DOI:** 10.3389/fcell.2015.00068

**Published:** 2015-10-30

**Authors:** Fernando A. Fierro, Adam J. O'Neal, Julie R. Beegle, Myra N. Chávez, Thomas R. Peavy, Roslyn R. Isseroff, José T. Egaña

**Affiliations:** ^1^Stem Cell Program, Department of Cell Biology and Human Anatomy, University of California, DavisDavis, CA, USA; ^2^Department of Biological Sciences, California State UniversitySacramento, CA, USA; ^3^Department of Plastic Surgery and Hand Surgery, University Hospital rechts der Isar, Technische Universität MünchenMunich, Germany; ^4^Facultad de Ciencias, FONDAP Center for Genome Regulation, Universidad de ChileSantiago, Chile; ^5^Department of Dermatology, University of California, DavisDavis, CA, USA; ^6^Institute for Medical and Biological Engineering, Schools of Engineering, Biological Sciences and Medicine, Pontificia Universidad Católica de ChileSantiago, Chile

**Keywords:** mesenchymal stem cells, scaffolds, wound healing, angiogenesis, hypoxia

## Abstract

Many therapies using mesenchymal stem cells (MSC) rely on their ability to produce and release paracrine signals with chemotactic and pro-angiogenic activity. These characteristics, however, are mostly studied under standard *in vitro* culture conditions. In contrast, various novel cell-based therapies imply pre-seeding MSC into bio-artificial scaffolds. Here we describe human bone marrow-derived MSC seeded in Integra matrices, a common type of scaffold for dermal regeneration (SDR). We show and measured the distribution of MSC within the SDR, where cells clearly establish physical interactions with the scaffold, exhibiting constant metabolic activity for at least 15 days. In the SDR, MSC secrete VEGF and SDF-1α and induce transwell migration of CD34^+^ hematopoietic/endothelial progenitor cells, which is inhibited in the presence of a CXCR4/SDF-1α antagonist. MSC in SDR respond to hypoxia by altering levels of angiogenic signals such as Angiogenin, Serpin-1, uPA, and IL-8. Finally, we show that MSC-containing SDR that have been pre-incubated in hypoxia show higher infiltration of endothelial cells after implantation into immune deficient mice. Our data show that MSC are fully functional *ex vivo* when implanted into SDR. In addition, our results strongly support the notion of hypoxic pre-conditioning MSC-containing SDR, in order to promote angiogenesis in the wounds.

## Introduction

Skin is the largest organ of the body and is responsible for several critical functions, such as control of body temperature and protection against external pathogens. Skin defects lead to the death of thousands of people per year and represent large costs for the health care system. In this regard, the development of scaffolds for dermal regeneration (SDR) has meant a significant breakthrough, especially for patients presenting large burns (Machens et al., 2000). However, the clinical benefit of these scaffolds in chronic wounds has been disappointing due to lack of induction of dermal regeneration and re-epithelialization. We and others have shown that SDR-mediated skin repair is greatly improved if SDR are “bio-activated” by pre-implantation of cells (Markowicz et al., 2006; Falanga et al., 2007; Egaña et al., 2009).

A very promising cell source for skin repair is mesenchymal stem cells/multipotent stromal cells (MSC), which, in many applications, do not contribute to regeneration through direct differentiation into dermal, epithelial, or endothelial cells (tissue replacement), but rather act as trophic mediators, releasing chemotactic, immune modulatory, and pro-angiogenic factors (Caplan and Dennis, 2006; Fu and Li, 2009). MSC are easy to isolate from various tissue sources, including bone marrow and adipose tissue (da Silva Meirelles et al., 2006), can be robustly expanded *ex vivo* and present low immunogenicity, allowing both autologous and allogeneic transplants (Uccelli et al., 2008). Albeit an overall elusive function *in vivo* due to the absence of specific MSC surface markers, MSC seem to correlate with pericytes (Crisan et al., 2008). MSC from the bone marrow are the primary source for osteo-progenitor cells and play a critical role in supporting hematopoietic stem/progenitor cells (Sacchetti et al., 2007). It has also been shown that direct intradermal injection of human MSC promote wound healing in diabetic mice (Kim et al., 2012; Shin and Peterson, 2013).

MSC that are expanded for *in vitro* studies are commonly cultured in polystyrene plastic flasks or plates. The capability to attach to plastic is indeed a major defining characteristic of MSC (Dominici et al., 2006). However, parameters such as the high stiffness of plastic strongly affect MSC cell fate (McBeath et al., 2004; Engler et al., 2006), suggesting that characteristics attributed to MSC will vary according to the specific *in vitro* culture conditions. For example, when MSC cultured in three-dimensional (3D) scaffolds are implanted into nude mice, they generate more abundant and homogenous bone as compared to MSC cultured as monolayers (Braccini et al., 2005). MSC cultured in 3D scaffolds respond to hypoxia by expressing higher levels of the stem cell markers Oct-4 and Rex-1, and maintain a higher colony forming unit (CFU-F) potential as compared to cells in normoxia (Grayson et al., 2006). Hypoxia also enhances the motility of MSC, improving their therapeutic potential on blood flow restoration, as shown using a murine hind limb ischemia model (Rosová et al., 2008). Finally, we have recently shown that hypoxic pre-conditioning induces metabolic changes in MSC that promote their retention after intramuscular injection into immune deficient mice (Beegle et al., 2015).

This development suggests that combining scaffolds for dermal regeneration (SDR) with MSC for the treatment of chronic skin ulcers and wound repair would be an ideal strategy. We recently compared incorporation of adipose-tissue derived MSC into different scaffolds. In those studies, parameters such as seeding efficiency, distribution, attachment, survival, metabolic activity, and release of paracrine signals where measured, where the best results were obtained with Integra™ matrices (Wahl et al., 2015). Integra™ Matrix Wound Dressing is a bilayer scaffold composed of type I bovine collagen and chondroitin-6-sulfate with a thin silicon layer. We have also shown that Integra™ SDR seeded with a mesenchymal cell line show a strong increase of new blood vessel formation, following a skin wound excision model in nude mice (Egaña et al., 2009). These results strongly support the strategy of “bio-activating” Integra™ SDR by pre-seeding it with MSC.

Here we show that bone marrow-derived MSC implanted in three dimensional SDR promote key features for wound repair applications, such as sustained viability, migration of hematopoietic/endothelial progenitor cells (H/EPC) and response to hypoxia, inducing release of pro-angiogenic signals *in vitro*. Most importantly, our results also suggest that pre-incubation with hypoxia increases the angiogenic potential of MSC/SDR *in vivo*.

## Materials and methods

### Cell isolation and culture

MSC were obtained from either bone marrow samples of healthy human donors who gave informed consent to the research protocols (approved by the institutional review board of the Technical University of Dresden) or purchased from Allcells (Alameda, CA). Mononuclear cell (MNC) fraction of bone marrow aspirates were obtained by density gradient centrifugation [Percoll (1.073 g/l) for 30 min at 700 × g] and plated in plastic culture flasks with MSC culture media (see below). After 3 days, non-adherent cells were removed by 2–3 washing steps with PBS. MSC culture media used for viability assays, secretion of VEGF and SDF-1α and migration assays was low glucose (1 g/L) Dulbecco's modified Eagle's medium (DMEM; Invitrogen, Carlsbad, CA) supplemented with 10% fetal bovine serum (FBS; Biochrom AG, Berlin, Germany). MSC culture media used for confocal imaging in SDR, analysis of distribution, hypoxia-induced angiogenic proteome array and *in vivo* studies was MEM-alpha (HyClone Thermo Scientific, Waltham MA) supplemented with 10% FBS (Atlanta Biologicals, Lawrenceville GA). In all cases, MSC from passages 2–6 where used for experimentation. All MSC are routinely characterized for immune phenotype (CD14^−^, CD34^−^, CD45^−^, CD73^+^, CD90^+^, CD105^+^, and CD166^+^) and differentiation potential into both adipocytes and osteoblasts (not shown).

For isolation of CD34^+^ cells, leukapheresis samples were obtained from healthy donors, whose stem cells were mobilized by administration of GM-CSF, following institutional approval. MNC were separated by density gradient [centrifugation over Percoll (1.073 g/l) 30 min at 700 × g]. CD34^+^ cells were isolated using anti-CD34 coated paramagnetic microbeads according to manufacturer instructions (Miltenyi, Begisch-Gladbach, Germany).

### Seeding MSC in SDR

For all experiments performed with MSC in SDR (Integra™ Matrix Wound Dressing; Plainsboro, NJ), the following protocol was used: Pieces of SDR matrices (10 mm diameter, approximate volume; 0.3 cm^3^) were dried with sterile gauze, placed in 24-well plates and 2.5 × 10^5^ MSC in 300 μl culture medium were dropped over the scaffold and quickly absorbed. After 30 min of incubation in the cell culture hood, 1 ml of culture media was added to each well.

### Imaging of SDR by scanning electron microscopy

For scanning electron microscopy of SDR, scaffolds were washed with PBS and fixed with 2% glutaraldehyde for 1 h at room temperature and then overnight at 4°C. SDR were dehydrated in a series of acetone (30–100%) and critical-point-dried in a CO_2_ system (Critical Point Dryer CPD 030, BAL-TEC GmbH, Witten, Germany). Samples were then sectioned and mounted on aluminum stubs and sputter-coated with gold (Sputter Coating Device SCD 050, BAL-TEC GmbH, Witten, Germany). Finally, samples were analyzed at 10 kV accelerating voltage in an environmental scanning electron microscope (XL 30; Philips, Eindhoven, The Netherlands).

### Imaging of MSC in SDR by laser scanning confocal microscopy

For the observation of MSC in SDR, the following protocol was followed: 3 days after seeding MSC in SDR, scaffolds containing cells were rinsed with PBS, fixed for 30 min with paraformaldehyde (3.7% paraformaldehyde, 0.1% Triton in PBS), and blocked in 2% BSA in PBS prior to incubation in phalloidin-texas red to stain actin and To-Pro3 to stain DNA. After washing, scaffolds were imaged using a confocal microscope to assess the number of cells that were seeded, the distribution of cells and their morphology. Three SDR were examined, and each of which was seeded using MSC derived from three different donors. Since Integra™ Matrix was ~1 mm in thickness, the matrices were sectioned, turned on their sides and z-section image series acquired (about 100 μm z-sect depth). Seven sections were imaged and analyzed so as to gather a representative sampling. Fluorescence imaging of cells upon and within the matrix were optimized so as to avoid spectral overlap since collagen was observed to auto-fluorescence in UV and FITC channels (emission ranging over 300–500 nm).

### Cell loading capacity and cell retention in SDR

MSC cultured in SDR for up to 15 days were incubated for 3 h in fresh medium containing 5 ng/ml of MTT (3-(4,5-dimethyl-2-Thiazolyl)-2,5-Diphenyl-2H-Tetrazolium bromide) (Sigma-Aldrich, Turkirchen, Germany). Then, medium was removed and replaced by 300 μl dimethyl sulfoxide (DMSO). After 15 min incubation, DMSO was removed and absorbance was measured at 570 nm to quantify formation of formazan blue, which is proportional to the number of living cells. Scaffolds without cells were used as negative control.

### VEGF and SDF-1α release from MSC in SDR

Two days after seeding MSC in SDR, medium was replaced with DMEM + 2% FBS. Then, every 48 h medium was removed and replaced with fresh medium. VEGF and SDF-1α concentrations were measured by ELISA according to manufacturer instructions (Quantikine ELISA kits, R&D systems, Minneapolis, MN). Scaffolds without cells were used as negative controls.

### Migration assay

2 × 10^5^ CD34^+^ cells in 100 μl RPMI were added into inserts of 5 μm-pore transwell plates (Corning Inc., Corning, NY) with conditioned media of MSC-containing SDR or empty SDR (control) in the lower compartment. After incubation for 4 h, inserts were removed and cells that migrated into the lower compartment were counted using Trypan blue exclusion dye and a hemocytometer. The effect of blocking the CXCR4 receptor was evaluated by pre-incubation of the cells for 2 h in media containing 100 μM AMD3100 (Sigma).

### Angiogenesis proteome array

MSC derived from three different donors were seeded in SDR as described above. After 24 h, medium was changed to MEM-alpha + 2% FBS and cells cultured for 48 h either under normal (21% O_2_) or hypoxic (3% O_2_) conditions. Next, media were collected and used in a Proteome Profiler Human Angiogenesis Array Kit, following manufacturer instructions (R&D Systems). Hypoxia was generated in an incubator at 37°C with 5% CO_2_ humidified atmosphere and dedicated oxygen level (3% O2), as established by replacement with Nitrogen injections.

### Endothelial cell migration in a wound excision model *in vivo*

All animal procedures were performed strictly adhering to protocols approved by the Institutional Animal Care and Use Committee at UC Davis. Two excisional wounds were generated bilaterally on the flanks of immune deficient NOD/SCID IL2Rγ –/– (NSG) mice, as previously described (Egaña et al., 2009; Schenck et al., 2014). Briefly, animals were placed under anesthesia (1.5–3% isoflurane), fur was shaved and an area of skin was removed on both the left and/or right flank of the animal using a 10 mm biopsy punch. Then, a 15 mm piece of surgical mesh (TiMESH Titanized Polymers, Nuernberg, Germany) was placed under the wound edge, thus covering the wound bed. Next, an 8–10 mm piece of SDR was placed over the wound bed. Each condition, (SDR alone or SDR containing MSC pre-incubated for 2 days in either 21 or 3% O_2_) was placed bilaterally into 3 animals for a total of 6 replicates. SDR were fixed at 6 points with skin glue (Histoacryl Topical Skin Adhesive, TissueSeal, Ann Arbor, MI), such that the wound edge overlapped 1–2 mm with the scaffold. Finally, wounds were covered with sterile gauze and adhesive tape and monitored daily.

After 14 days, animals were euthanized and SDR with surrounding tissue processed for histological analysis. Samples were transferred to a blocking solution (2% BSA in PBS) at room temperature for 2 h. Then, tissues were blotted dry with gauze and incubated in PBS containing 0.5% Triton X-100 and a 1:75 dilution of Biotinylated GSL I—isolectin B4 (Vector Laboratories, Burlingame, CA), was applied at room temperature for 1 h with gentle agitation on a platform shaker. GSL I—isolectin B4 has been shown to recognize terminal α-galactosyl residues found on the glycoprotein laminin secreted by endothelial cells while forming the basal lamina of blood vessels (Benton et al., 2008). After washing 3 times for 5 min each with PBS containing 0.5% Triton X-100, Dylight 649-streptavidin secondary antibody (Vector Labs) at a 1:150 dilution was applied at room temperature for 1 h with shaking. After 3 additional washes with PBS + 0.5% Triton X-100 and a final wash with PBS, samples were mounted in VectaShield containing DAPI (Vector Labs). Imaging was done on an Olympus FluoView FV10i confocal microscope. Each sample was cut into up to four sections and each cut was placed on its cross-section for imaging. At least 35 images per condition were analyzed and averaged for the quantification of endothelial cell infiltration.

### Statistical analysis

All assays were repeated in at least three independent experiments. Results are expressed as averages with the standard error of the mean (SEM) as error bars, unless otherwise stated. One-Way ANOVA or Student's *t*-tests were used to compare samples, where *p* < 0.05 was considered significantly different.

## Results

**Figure 1:**
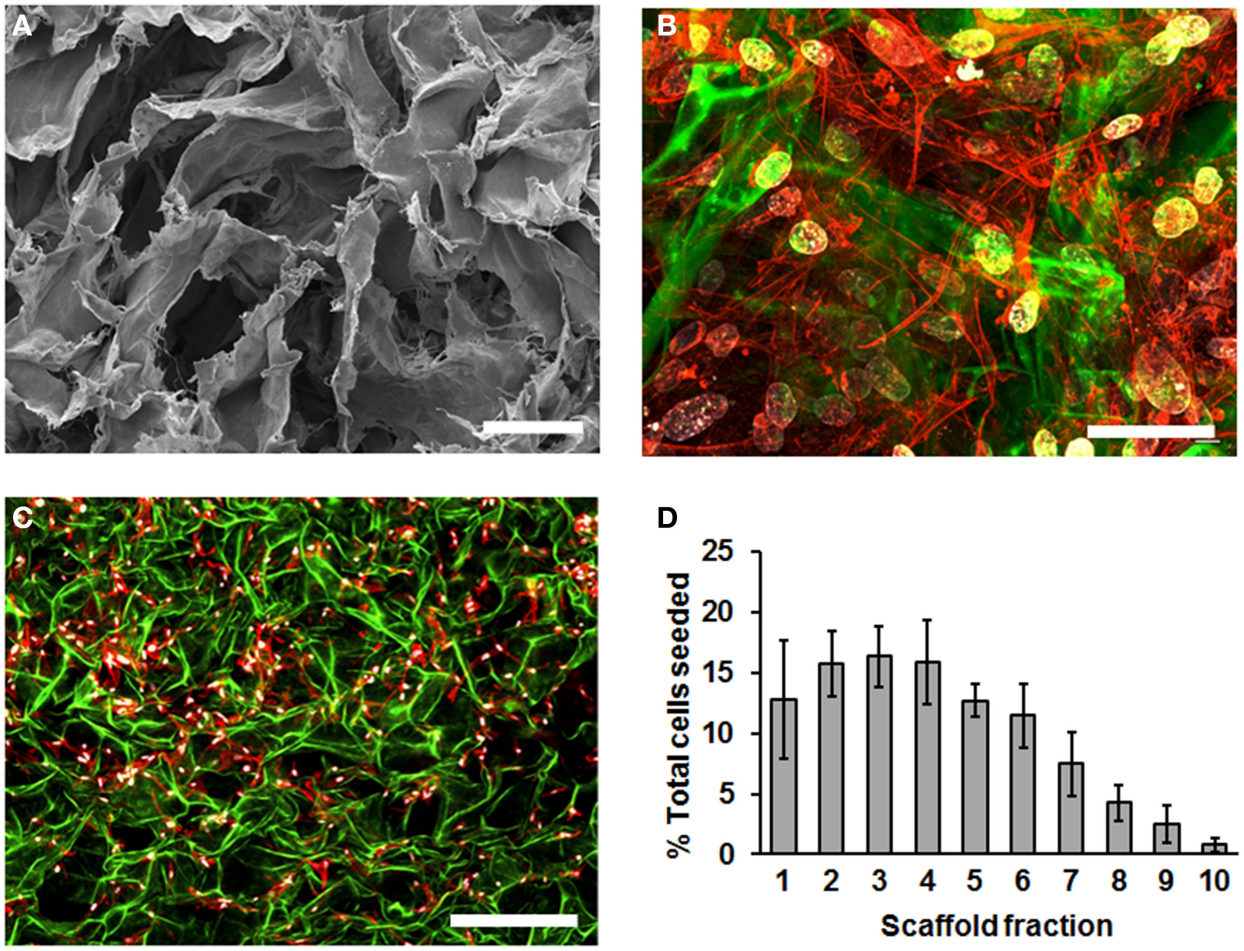
**SDR topology and distribution of MSC in them. (A)** SDR as observed using scanning electron microscopy (SEM). SDR has a randomly folded laminar structure, which generates large spaces of 50–200 mm in diameter. **(B–D)** 2.5 × 10^5^ MSC were seeded in SDR, cultured for 3 days to allow maximum attachment and spreading, and fixed for staining as follows: Nuclei were stained with DAPI (blue), actin cytoskeleton is labeled with TRITC-coupled phalloidin and auto-fluorescence of SDR is shown in green. Although cell distribution appears relatively homogenous within the scaffold surface, 85% of cells were found concentrated in the upper half of the scaffold **(C,D)**. To quantify cell distribution, cell nuclei were counted within each z-section image and their distribution tabulated with respect to the scaffold fraction. Also using this z-stack analysis, we inferred an average seeding efficiency of 92%. Scale bar represents 0.1 μm in **(A)**, 50 μm in **(B)**, and 500 μm in **(C)**.

To characterize MSC cultured in SDR, we first used scanning electron microscopy (SEM) to inspect the scaffold. We observed that the SDR has a randomly folded laminar structure, which generates large spaces of 50–200 μm in diameter (Figure 1A). To evaluate how MSC distribute within this scaffold, we seeded the MSC in SDR and cultured them for 3 days to allow maximum attachment and spreading within the scaffold. Then, samples were fixed and examined using confocal imaging. We counted cell nuclei within each z-section image and tabulated their distribution with respect to the scaffold fraction. Although cell distribution appeared relatively homogenous within the scaffold (Figure 1B), 85% of cells were found concentrated in the upper half of the scaffold (Figures 1C,D). Also using this z-stack analysis, we inferred an average seeding efficiency of 92%. MSC were observed to be well spread throughout the matrix as evidenced by actin staining (Figures 1B,C).

**Figure 2:**
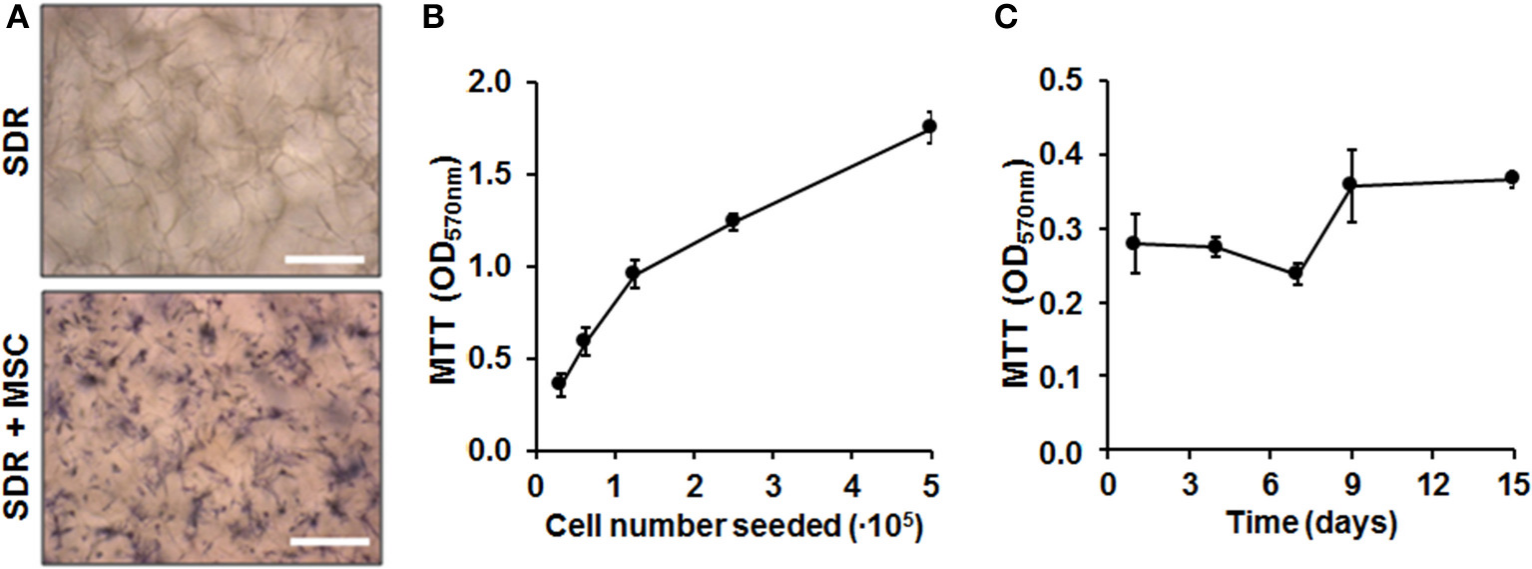
**Cell load capacity and cellular viability within SDR matrices. (A)** MTT assay on empty- and MSC-containing SDR matrix demonstrates specific formazan formation by cells (dark blue). **(B)** MSC were seeded in 0.13 cm^2^ pieces of SDR matrix and cultured for 24 h to allow maximum spreading and adhesion of cells. Then, scaffolds with cells were incubated with MTT substrate to determine relative cellularity based on formazan blue formation. **(C)** MSC in SDR matrices shows relatively constant MTT hydrolysis for up to 15 days. Scale bars represent 200 μm.

Next we quantified cell load-capacity of SDR using a MTT assay. First, we demonstrate that there was no unspecific labeling with MTT substrate in empty scaffolds. In contrast, MSC-containing scaffolds show cells turning a dark blue color, which can be clearly visualized using microscopy (Figure 2A). Next, we seeded increasing doses of MSC and concluded that the SDR supports high cellularity, with the highest concentration tested equated to 3.6 × 10^6^ cells per 1 cm^2^ (Figure 2B). To evaluate whether MSCs were viable and if they proliferate within the SDR matrix, we cultured the cells for up to 15 days. As shown in Figure 2C, we found constant formazan blue formation, suggesting that MSC are viable and metabolically stable within the scaffold for at least 15 days, but show limited expansion.

**Figure 3:**
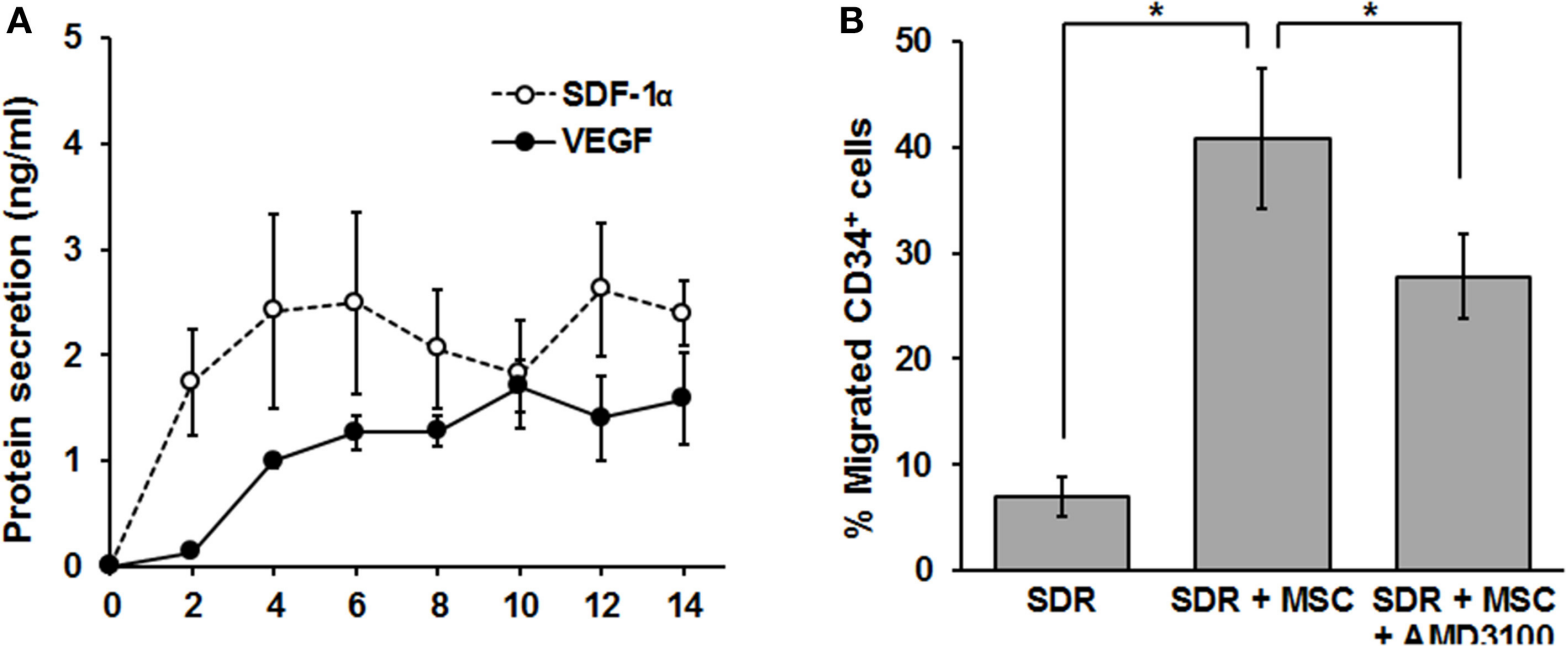
**MSC-containing SDR secrete VEGF, SDF-1α, and induce migration of CD34^+^ H/EPC ***in vitro***. (A)** MSC-containing SDR were incubated with medium changes every second day. At every medium change, supernatants were collected, and VEGF and SDF-1α protein levels measured by ELISA. Average of MSC from 3 different donors are shown. **(B)** Supernatant of SDR (control) and SDR + MSC were collected after 48 h incubation and tested for CD34^+^ H/EPC migration in a transwell assay, as described in the Materials and Methods Section. In addition, AMD3100 (100 ng/ml) was added to the SDR + MSC condition during the transwell assay. Average of CD34^+^ of 3 different donors using MSCs from 2 different donors are shown. ^*^p < 0.05.

In the following set of experiments, we determined whether the SDR could be “bio-activated” with MSC in terms of secreting functionally relevant levels of chemotactic and angiogenic signals. The development of new vasculature during the tissue repair process is driven by two major mechanisms: angiogenesis and vasculogenesis (Carmeliet, 2000). While the latter implies the recruitment of new endothelial progenitor cells (EPC), angiogenesis is commonly defined as the extension of pre-existing blood vessels and is therefore dependent on the proliferation and organization of endothelial cells (Carmeliet, 2000). Vascular remodeling (vasculogenesis) relies on the recruitment of CD34^+^ EPC (Asahara et al., 1997). Since EPC mobilization and homing to the site of injury depends on the CXCR4/SDF-1α axis (Tachibana et al., 1998; Moore et al., 2001), we tested whether MSC-containing scaffolds could produce and release significant levels of SDF-1α. For this, MSC were seeded in SDR matrix, medium was changed every second day and SDF-1α protein levels were measured in the collected supernatant by ELISA. As shown in Figure 3A, SDF-1α levels are low for the first 2 days after seeding MSC in SDR. However, constant levels (ranging from 1.3 to 1.7 ng/ml) of SDF-1α were detected from days 4 to 14. Most importantly, MSC-containing scaffolds are capable of inducing migration of CD34^+^ cells (comprising both hematopoietic- and endothelial- progenitor cells (H/EPC) in a transwell-migration assay, demonstrating that the chemotactic signals released are fully functional. Furthermore, the migration of CD34^+^ cells was partially inhibited by the CXCR4 antagonist AMD3100, strongly suggesting that the migration of CD34^+^ cells is at least partly due to SDF-1α released by MSC-containing SDR (Figure 3B).

**Figure 4:**
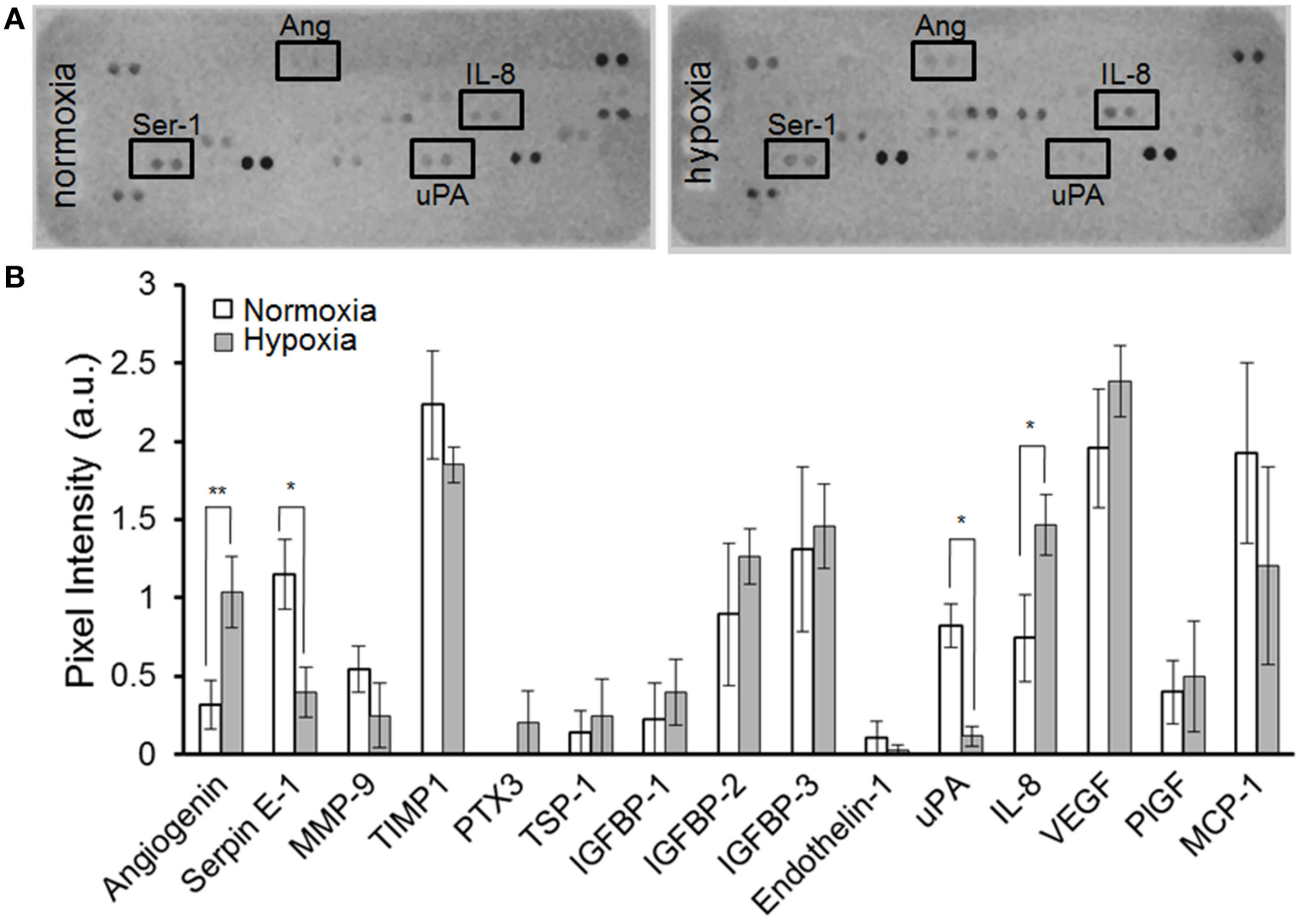
**MSC-containing SDR secrete angiogenic factors and respond to hypoxia**. MSC-containing SDR were cultured under normoxic or hypoxic conditions for 2 days and supernatant collected for angiogenic factors as described in Section Materials and Methods. **(A)** Representative angiogenesis-proteome array. **(B)** Quantification of angiogenesis proteome array, using MSC from 3 different donors. ^*^p < 0.05; ^**^p < 0.005.

As shown in Figure 3A, MSC-containing SDR also release constant levels of the angiogenic signal vascular endothelial growth factor (VEGF) (2.2+0.5 ng/ml). In addition, we tested whether MSC are able to respond to hypoxia when seeded in SDR. For this, seeded scaffolds were cultured for 48 h in the presence of either 21% (normoxia) or 3% O_2_ (hypoxia). As shown in Figures 4A,B, several angiogenic signals could be detected in supernatants of MSC-seeded SDR. Among them, angiogenin (Ang), and interleukin 8 (IL-8) were significantly up-regulated during hypoxia, while serpin-1 and urokinase-type plasminogen activator (uPA) protein levels were strongly inhibited by hypoxia.

**Figure 5:**
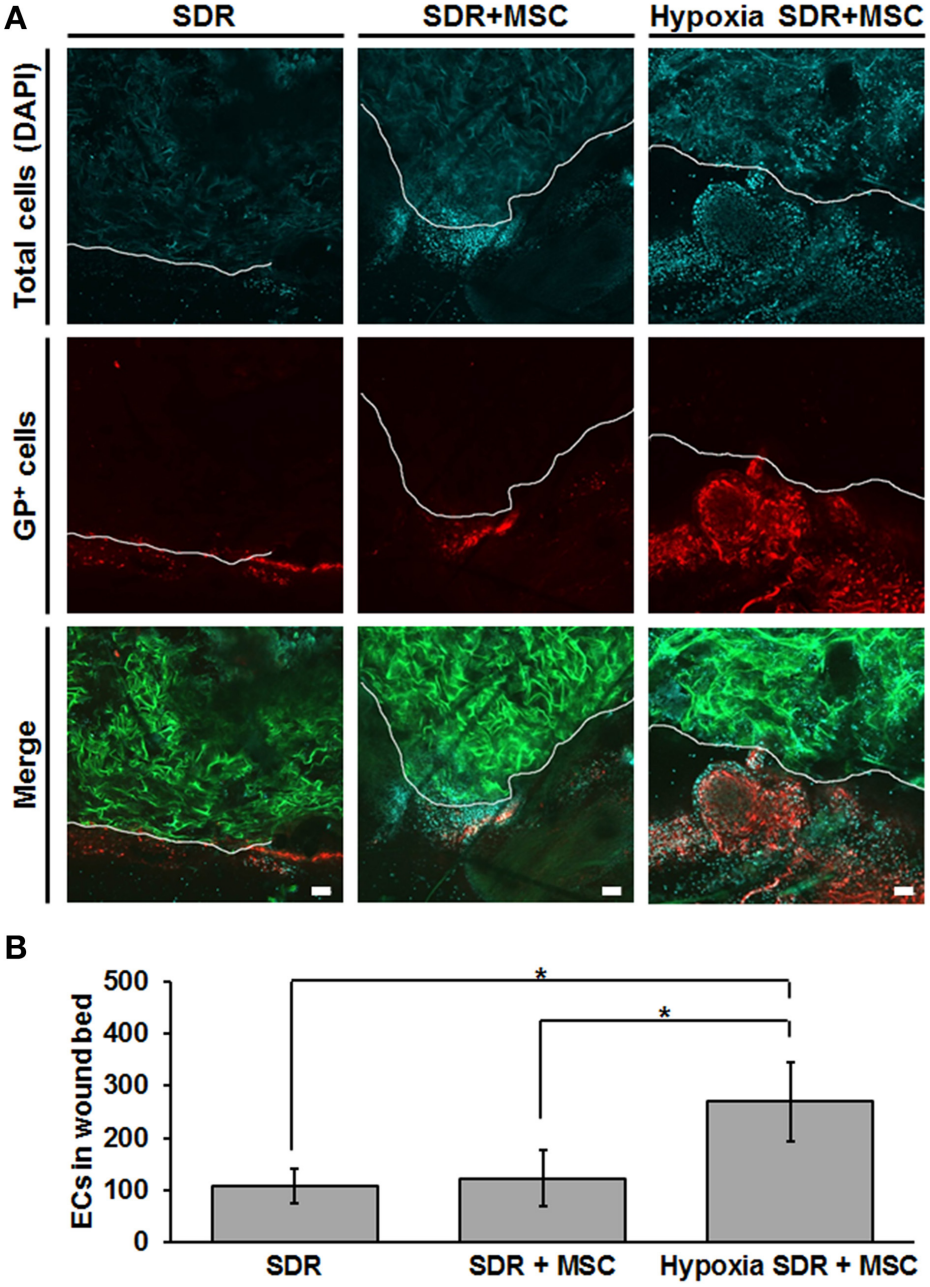
**Hypoxic pre-conditioning of MSC-containing SDR favors high cellular infiltration ***in vivo*****. MSC-containing SDR were cultured under normoxic or hypoxic conditions for 2 days and then implanted into immune deficient NSG mice, following a skin excision procedure. After 2 weeks, animals were euthanized and scaffold and surrounding tissue were analyzed using laser scanning confocal microscopy. **(A)** Green shows the SDR (due to auto-fluorescence of collagen), blue shows DAPI staining of cell nuclei, and red shows the staining of GSLI isolectin B4 which binds to the terminal α-D-galactosyl residues of the glycoprotein laminin (GP) expressed by endothelial cells within the basal lamina of neovascular structures. The white line indicates the border of the scaffold and surrounding tissue. **(B)** Quantification of endothelial cells (ECs), as determined by the average number of GP-positive cells within the first 200 μm of SDR near wound edge. Scale bar represents 50 μm. ^*^p < 0.05.

Finally, we tested whether MSC-seeded SDR pre-incubated for 48 h in hypoxia would exert a functional effect *in vivo*. For this we generated bilateral excisional full skin defects in mice and implanted in each either SDR alone or MSC-containing SDR that were pre-incubated for 2 days in either normoxic or hypoxic conditions. Figure 5 shows both representative images and the quantification of endothelial cells in the wound area that were secreting the specific glycoform of laminin found in the basal lamina of blood vessels, 14 days after surgery. Previous studies have shown that GSLI-isolectin B4 labels only the basal lamina of blood vessels secreted by endothelial cells within the dermal layers (Brabec et al., 1980; Benton et al., 2008). While no significant differences were found between SDR alone and MSC-containing SDR in normoxia, pre-incubation for 2 days in hypoxia had a highly significant effect promoting endothelial cell migration into wound edge, suggesting that hypoxic pre-conditioning is essential for promoting blood vessel formation in MSC-containing SDR.

## Discussion

The reparative potential of skin is limited because wounds are typically replaced by scar formation, which restores tissue integrity, but not full functionality. This is critical in massive (e.g., burns) and specific (e.g., neck, hands, elbows) skin injuries where functional tissue is required. In this context, SDR have been used as templates to favor a skin regeneration process. However, an important limitation in efficacy of SDR to induce tissue regeneration is the lack of proper vascularization. This limitation could be circumvented by “bio-activating” the SDR with MSC, which are able to induce angiogenesis and recruit H/EPC via secretion of paracrine factors. Nevertheless, these properties of MSC have been studied *in vitro* under standard culture conditions, where cells are grown as monolayers adhered to plastic. In contrast, little is known about their performance in 3D structures. SDR provide a very different cell culture condition, generating several local microenvironments and gradients, which may influence the exocrine profile of MSC, as well as their proliferation and survival, among others.

Ultra-structural characterization of the SDR used in this study (Integra™ Matrix Wound Dressing) revealed 50–200 μm diameter pores within the scaffold. Of note, during the preparation process, the SDR was dried, possibly altering the fine structure morphology of the matrix. Commercially, this SDR is stabilized with glutaraldehyde and stored in sodium phosphate buffer. However, our images show a structure that allows a homogenous distribution of MSC, as observed by confocal microscopy. Of note, a key step in our protocol is the seeding process: if MSC are seeded into wet scaffolds, cells stay attached in higher densities at the surface of the SDR (not shown), presumably due to a high affinity of MSC to attach to collagen (Cool and Nurcombe, 2005). In contrast, slightly drying the SDR with subsequent addition of highly concentrated MSC (~1000 cells/μl) promotes absorption of the cells into the SDR, favoring the penetration of cells into the scaffold. Addition of more culture media 20–30 min later ensures optimal MSC culture conditions. Our distribution analysis reveals that 85% of cells are concentrated in the upper half of the scaffold, suggesting that for clinical applications, the MSC-containing SDR should be placed with the upper side facing toward the wound, in order to favor the interaction of MSC with surrounding tissue. Alternatively, MSC could be seeded from both sides to further increase the total amount of cells incorporated. Direct interaction of MSC in the SDR was demonstrated as cells attached and spread, acquiring characteristic fibroblastic morphology. The adhesion of MSC is critical, since detachment of cells induces programmed cell death, a process known as anoikis (Frisch and Francis, 1994).

The notion that MSC attached to the scaffold remain viable is supported by detection of constant formazan formation. Our results also suggest that MSC may not extensively proliferate under these culture conditions. This is further supported by the steady levels of secreted SDF-1α and VEGF reached by 2–4 days after seeding. The proliferation of MSC could be limited due to a rather low volume of culture media added to each scaffold/well, limiting nutrient availability. Alternatively, integrin-mediated adhesion of MSC to the matrix may inhibit cell proliferation (Giancotti and Ruoslahti, 1999). The constant release of factors such as VEGF and SDF-1α could possibly have an important impact on the regeneration process. VEGF is a potent mitogen for endothelial cells and induces endothelial cell migration, sprouting, and survival (Ozawa et al., 2004). The CXCR4/SDF-1α axis is essential for stem cell homing and mobilization into damage tissues (Tachibana et al., 1998; Peled et al., 1999; Ceradini et al., 2004). Of note, SDF-1α has been shown to improve wound healing in diabetic mice (Badillo et al., 2007).

Here we show that MSC seeded in SDR respond to hypoxia, which alters their expression of angiogenic factors. We found that MSC in SDR under hypoxia increase Ang and IL-8 secretion. This effect has been reported in various cell types, including MSC cultured under standard conditions (Hung et al., 2007; Potier et al., 2007). Ang is an important RNAse that promotes neovascularization. When new vessels are required, Ang is incorporated into endothelial cells and transferred to their nuclei. There, it stimulates rRNA transcription, a rate-limiting step in ribosome biogenesis, protein translation, and cell growth (Wiedlocha, 1999; Tello-Montoliu et al., 2006). It has also been shown that angiogenin is necessary for angiogenesis induced by factors such as VEGF (Kishimoto et al., 2005). Expression of the chemokine IL-8 directly correlates with neovascularization (Yoneda et al., 1998) by stimulating both proliferation and migration of endothelial cells (Brat et al., 2005). We hypothesize that the observed reduction of PAI-1 and uPA under hypoxia are inter-related events, because interaction of PAI-1 with the uPA/uPA-receptor complex induces internalization of the ternary complex uPA-R/uPA/PAI-1, resulting in degradation of uPA and PAI-1, while uPA-R is recycled to the cell surface (Harbeck et al., 2004). According to our angiogenesis array, hypoxia only induced a modest increase in VEGF levels in MSC in SDR. However, using ELISA, we and others have observed that expression of VEGF is significantly increased in MSC under hypoxia (not shown). This is most likely due to limited sensitivity of the angiogenesis array, where VEGF detection approached saturation levels (maximal pixel intensity). The angiogenesis array is only a semi-quantification based on pixel intensity of the dots, rather than colorimetric optical density, with quantification based on a standard curve, as used in ELISAs.

The exocrine effects of MSC could have tremendous impacts on the development of new strategies to enhance vascularization and tissue regeneration in tissue engineering approaches. The use of MSC seeded scaffolds should be studied in massive or chronic wounds in *in vivo* settings. Indeed, our *in vivo* studies strongly support the notion that hypoxic pre-conditioning favors the infiltration of endothelial cells into the wound bed that are in the early stages of neovascularization. The mechanism, however, remains unclear. One possibility is that hypoxic pre-conditioning enhances the survival of MSC after implantation, hence a greater number of endothelial cells is favored by increased presence of MSC (Beegle et al., 2015). Alternatively, hypoxia increases the secretion of angiogenic signals (as shown *in vitro*) that promote endothelial cell migration. These and other hypotheses need future experimental validation. In addition, future studies need to directly address the effect of hypoxic MSC-containing SDR in wound closure.

This work underlines the relevance of studying MSC not only under standard culture conditions, but also in environments that more closely mimic their potential clinical application. Our results suggest that seeding MSC into SDR potentially “bio-activates” the material to enhance the regenerative process. Finally, we show that hypoxic-preconditioning of MSC-containing SDR has a strong positive impact promoting endothelial cell infiltration toward the wound bed, possibly contributing the wound repair process.

## Grant support

This project was funded by Early Translational Grant TR2-01787 from the California Institute for Regenerative Medicine (CIRM) and the German Federal Ministry of Education and Research (BMBF). The publication of this work was supported by the German Research Foundation (DFG) and the Technische Universität München within the funding programme Open Access Publishing. All authors declare no conflicts of interest regarding this publication or any information related to it.

### Conflict of interest statement

The authors declare that the research was conducted in the absence of any commercial or financial relationships that could be construed as a potential conflict of interest.
